# Immunohistochemical Analysis of Intestinal and Central Nervous System Morphology in an Obese Animal Model (*Danio rerio*) Treated with 3,5-T2: A Possible Farm Management Practice?

**DOI:** 10.3390/ani10071131

**Published:** 2020-07-03

**Authors:** Roberta Imperatore, Lea Tunisi, Isabella Mavaro, Livia D’Angelo, Chiara Attanasio, Omid Safari, Hamidreza Ahmadniaye Motlagh, Paolo De Girolamo, Luigia Cristino, Ettore Varricchio, Marina Paolucci

**Affiliations:** 1 Department of Science and Technology (DST), University of Sannio, 82100 Benevento, Italy; rimperatore@unisannio.it (R.I.); etvarric@unisannio.it (E.V.); 2 Endocannabinoid Research Group, Institute of Biomolecular Chemistry-National Research Council (ICB-CNR), 80078 Pozzuoli, Italy; lea.tunisi@unina.it (L.T.); luigia.cristino@icb.cnr.it (L.C.); 3 Department of Veterinary Medicine and Animal Productions, University of Naples Federico II, 80137 Naples, Italy; isabella.mavaro@unina.it (I.M.); livia.dangelo@unina.it (L.D.); chiara.attanasio@unina.it (C.A.); degirola@unina.it (P.D.G.); 4 Center for Advanced Biomaterials for Healthcare, Istituto Italiano di Tecnologia, 80125 Naples, Italy; 5 Department of Fisheries, Faculty of Natural Resources and Environment, Ferdowsi University of Mashhad, Mashhad 9177948974, Iran; omid_safary@yahoo.com (O.S.); ahmadnia@um.ac.ir (H.A.M.); 6 Institute of Food Science, National Research Council (ISA-CNR), 83100 Avellino, Italy

**Keywords:** 3,5-diiodo-L-thyronine, zebrafish, diet-induced obesity, intestinal inflammation, brain inflammation

## Abstract

**Simple Summary:**

The obesity induced by overconsumption of nutrients leads to systemic inflammation and alters metabolic homeostasis by acting on central nervous system and peripheral tissues such as intestine. The 3,5-diiodo-L-thyronine (3,5-T2) is well-known for its positive role on fat mass and lipid metabolism, and at date, it is widely used as a drug for the treatment of obesity. However, the safe and effective dose as well as the possible adverse effects of this molecule have not been sufficiently explored. In this study, we analyzed the role of 3,5-T2 in regulating central and peripheral inflammation in diet-induced obese (D.I.O.) model of zebrafish. We found that 3,5-T2 sustained the intestinal alteration caused by D.I.O., as indicated by the high levels of pro-inflammatory cytokines, accompanied by a significant effect of 3,5-T2 on body weight and central inflammation in D.I.O. zebrafish. Therefore, the suggested potential use of 3,5-T2 to contrast obesity should be viewed with caution. We conclude that the zebrafish model can help to better understand the fundamental beneficial and side effects of 3,5-T2, which is of great importance to define the possible use of this metabolite of thyroid hormones as a drug in different diseases including obesity.

**Abstract:**

The 3,5-diiodo-L-thyronine (3,5-T2) is an endogenous metabolite of thyroid hormones, whose administration to rodents fed high-fat diet (HFD) prevents body weight increase and reverts the expression pattern of pro-inflammatory factors associated to HFD. The diet-induced obese (D.I.O.) zebrafish (Danio rerio) has been recently used as an experimental model to investigate fundamental processes underlying central and peripheral obesity-driven inflammation. Herein, we aim to understand the role of 3,5-T2 in regulating central and peripheral inflammation in D.I.O. model of zebrafish. 3,5-T2 (10 nM and 100 nM) was administered with the obesity-inducing diet (D.I.O. with 3,5-T2) or after 4 weeks of obesity-inducing diet (D.I.O. flw 3,5-T2). 3,5-T2 significantly increased the body weight and serum triglyceride levels in D.I.O. zebrafish in both conditions. Moreover, 3,5-T2 sustained or increased inflammation in the anterior (AI) and mid (MI) intestine when administered with the obesity-inducing diet, as indicated by the immunoexpression of the inflammatory markers tumor-necrosis factor-α (TNFα), cyclooxygenase 2 (COX2), calnexin, caspase 3, and proliferating cell nuclear antigen (PCNA). On the contrary, when 3,5-T2 was administered after the obesity-inducing diet, partly reverted the intestinal alteration induced by D.I.O. In addition, brain inflammation, as indicated by the increase in the activation of microglia, was detected in D.I.O. zebrafish and D.I.O. treated with 3,5-T2. These findings reveal that the effects of 3,5-T2 on fish intestine and brain can deviate from those shown in obese mammals, opening new avenues to the investigation of the potential impact of this thyroid metabolite in different diseases including obesity.

## 1. Introduction

Abnormal or excessive fat accumulation are generally accompanied by low chronic central and peripheral [1,2,3]. One of the main factors contributing to the development of obesity is the consumption of a high-fat diet (HFD) [1,4], characterized by high leptin levels in humans [5], mice and zebrafish (Danio rerio) [6,7,8] and increase in tumor necrosis factor α (TNF-α) and interleukin 6 (IL-6) levels [9]. Such pro-inflammatory factors are released into the blood stream contributing to the development of a chronic low-grade peripheral and central inflammatory status [10,11]. Many of the pathological conditions related to obesity derive from the impairment of mitochondrial respiration [12]. Since obesity causes serious pathologies, such as diabetes, hypertension, and dyslipidemia [11], its treatment is essential to prevent their development. Effective anti-obesity drugs able to counteract the excess of body adiposity without undesirable side effects are still needed. The use of thyroid hormones has been recently suggested to reduce weight and lipid accumulation. The 3,5-diiodo-L-thyronine (3,5-T2), an endogenous metabolite of thyroid hormones, has been identified as a biologically active iodothyronine [13,14,15], which acts as an allosteric regulator of cytochrome oxidase (COX) activity, and regulates mitochondrial activity and respiration [16], leading to a rapid increase in mitochondrial oxygen consumption [15,17]. Through these actions, 3,5-T2 seems to be able to revert the proinflammatory pattern activated in rats by HFD [18], playing important metabolic activities in HFD-fed rodents [19]. Moreover, chronic administration of 3,5-T2 (250 μg/100 g BW for 14 or 28 days i.p.) in diet-induced obese mice has beneficial effects on adiposity, serum leptin, and energy expenditure [20]. However, high levels of 3,5-T2 decrease body weight and blood glucose in obese mice, but induce thyrotoxicosis [21]. 3,5-T2 hypolipidemic effects have been studied in several animal models and it is reported that the effects of 3,5-T2 on metabolic efficiency are conserved across species [19,22]. However, little is known about the potential role of 3,5-T2 in the central and peripheral inflammation. Many studies are in progress to better understand whether 3,5-T2 can be a potential anti-obesity agent, but also to define its time- and dose-dependent activity, as well as the possible occurrence of undesirable side effects [16,19].

Zebrafish has been recognized as an excellent animal model to investigate the fundamental processes underlying human metabolic and inflammatory diseases, because of its similarity to mammals in terms of gut and brain functions as well as immunity related genes [23]. In zebrafish, as in mammals, the consumption of HFD induces general inflammation [3]. Diet-induced obese (D.I.O.) zebrafish model shows common pathophysiological pathways with obese mammalians, suggesting that zebrafish can be used as obesity model. Studies performed both in rodents and zebrafish have shown that the prolonged consumption of HFD leads to intestinal inflammation [1,2,3]. As in humans and rodents, also in zebrafish obesity-induced inflammation involves the activation of inflammatory mediators such as chemokines and cytokines (TNFα and interleukins) which are also highly conserved between zebrafish and mammals [3].

Despite the anatomical differences, the thyroid system is highly conserved in teleosts [24,25]. Indeed, several studies reported an important role of 3,5-T2 in regulating the development, growth, and metabolism in fish. Recently, Olvera et al. [26] showed that 3,5-T2 regulates genes involved in cell signaling and transcriptional pathways in the brain of tilapia (Oreochromis niloticus), while Little [27] and Navarrete-Ramirez [28] revealed that 3,5-T2 regulates thermal acclimation in zebrafish and growth in tilapia, respectively.

Considering that HFD is a major cause of systemic inflammation, D.I.O. zebrafish has been confirmed as a reliable animal model to identify putative pharmacological targets for the treatment of obesity and 3,5-T2 has been proposed to reduce obesity-induced inflammation, in this work we investigate the effect on peripheral and central inflammation of supraphysiological 3,5-T2 administration in the D.I.O. zebrafish model. In particular, to study the inflammation pathway we analyzed the gut morphology and the expression of the main inflammatory markers together with the related brain microglia activation. In addition, we describe the side effects of 3,5-T2 administration, providing useful information concerning the potential pharmacological use of this molecule.

## 2. Materials and Methods

### 2.1. Animals

Animals were housed under standard conditions of photoperiod (14:10 Light/Dark; ZT0, 9 Anti Meridian) and temperature (28 °C) [29]. The current study was carried out in the Aquaculture Lab of the Ferdowsi University of Mashhad (FUM). This project was approved by FUM animal ethics committee. Fish used in this study were treated in accordance with the European Commission recommendation 2007/526/EC and 2010/63/UE on revised guidelines for the accommodation and care of animals used for scientific purposes. All efforts were made to minimize fish suffering. Zebrafish did not receive medical treatment prior or during the experience. No deaths occurred before the experiment endpoint.

### 2.2. Zebrafish Feeding and Treatment

In feeding experiments, adult male zebrafish (0.35 ± 0.05 g) were divided into four dietary groups (n = 10 fish per group). (1) Control group: zebrafish fed once a day for 4 weeks with peeled Artemia salina cysts (22% fat, 44% proteins, 16% carbohydrates; Aqua Schwarz) in a weight-maintaining amount (5 mg Artemia per fish) (ctrl). (2) D.I.O. zebrafish group (diet-induced obesity): zebrafish made obese by feeding for 4 weeks with the same amount of Artemia salina of the control group, but three times a day (D.I.O.). (3) D.I.O. zebrafish followed by 3,5-T2 group: zebrafish made obese by 4 weeks of diet-inducing obesity and then fed with the same amount of Artemia salina of the control group adding 3,5-diiodothyronine (3,5-T2) (Sigma Aldrich) for 4 weeks (D.I.O. flw 3,5-T2). 3,5-T2 was added to the water at two final concentrations (10 nM and 100 nM). (4) D.I.O. zebrafish fed with 3,5-T2 group: zebrafish fed for 4 weeks with diet inducing obesity and at the same time treated with 3,5-T2 added to the water at two final concentrations (10 nM and 100 nM) (D.I.O. with 3,5-T2). Supraphysiological doses of 3,5-T2 were used according to Garcia et al. [30,31]. The addition of 3,5-T2 to the water was performed considering that the use of hydrophobic drugs by immersion provide an efficient, non-invasive, and minimally stressful mean of chronic administration in aquatic vertebrates [31]. The water was changed every day at 9:00 a.m. and 3,5-T2 was added. The efficacy of the administration of iodothyronine by immersion has been reported by Garcia and colleagues [30,31]. The D.I.O. feeding protocol used in this study was adapted from a previous study by Mania et al. [32]. After the trial, the zebrafish were fasted overnight and then suppressed. For every treatment, both the brain and the intestine from five fish were collected for histological analysis and immunohistochemistry. The 100-nM 3,5-T2 concentration showed toxicity with high mortality, therefore its administration was suspended.

### 2.3. Body Weight Measurement

Zebrafish body weight was determined by a precision analytical scale. Body weight (n = 10 animals/diet) was recorded at the beginning (week 0) and at the end (week 4 or 8) of the experiment. Specimens were fasted for 24 h and then suppressed.

### 2.4. Serum Lipid Parameters

At the end of the feeding experiments, blood was collected from 24-h-fasted zebrafish of all dietary groups (n = 10 animals/diet). For analyses of serum triglycerides, blood was collected by tail ablation. Serum was obtained by centrifugation at 2500× *g* for 10 min at 4 °C. Serum of three zebrafish was pooled for detection. Total triglycerides were measured using enzymatic colorimetric assays (BioAssay Systems, San Jose, CA, USA) following the manufacturer’s instructions. 

### 2.5. Morphological Analysis

Intestine samples were promptly fixed in 4% formalin in 0.01 M phosphate-buffered saline (PBS) pH 7.4 for no longer than 24 h at 4 °C, dehydrated in a graded series of ethanol, cleared with xylol, and embedded in paraffin. Samples were cut in 7 μm sections using a microtome [33]. For morphological analysis, anatomic comparable sections of intestinal bulb, herein indicated as anterior- (AI) and mid- (MI) intestine were deparaffinized with xylol and stained with hematoxylin-eosin (H&E). Histological sections were examined under light microscopy with a Leica DMI6000 equipped with Leica DFC340 cooled digital CCD camera (Leica Microsystems, Buccinasco MI, Italy) to obtain microscopic images at 20× magnification. To count the number of the goblet cells, anatomically comparable sections of AI and MI were stained with Alcian blue (1 g of Alcian blue, pH 2.5, 3 mL/L of acetic acid, and 97 mL of distilled water) for 1 h. Thereafter, the slides were rinsed in tap water for 10 min, oxidized in periodic acid (5 g/L) for 5 min, rinsed in lukewarm tap water for 10 min, and dehydrated in alcohol and clarified in xylol. Histological sections were examined by using the same microscope mentioned before at 10× magnification. In order to estimate the number of goblet cells, sections (n = 5 animal per treatment; n = 3 pairs of sections/animal, each section selected at a 50 μm distance to avoid counting the same cells) were analyzed by two independent operators blinded to the experimental protocol. For each section AI and MI were divided into three regions and goblet cells were counted in each of them.

### 2.6. Single Immunohistochemistry

For immunohistochemistry, the deparaffinized anatomic comparable sections of AI and MI were stained by the avidin-biotin immunohistochemical technique. The following antibodies were used: monoclonal antibodies raised in mouse against tumor-necrosis factor-α (TNFα) (code ab1793, Abcam, Cambridge, UK), polyclonal antibodies raised in rabbit against cyclooxygenase 2 (COX2) (code 69720, NovaTeinBio, Woburn, MA, USA), polyclonal antibodies raised in rabbit against calnexin (code NB100-1965, Novus Biologicals, Centennial, CO, USA), polyclonal antibodies raised in rabbit against caspase 3 (code ab13847, Abcam, Cambridge, UK), and monoclonal antibodies raised in mouse against proliferating cell nuclear antigen (PCNA) (code ab29, Abcam, Cambridge, UK). The sections were incubated for 5 min in 0.1% H_2_O_2_ to inactivate the endogenous peroxidase activity and then incubated for 30 min with 10% normal goat serum (NGS) (Vector Laboratories, Burlingame, CA, USA) in 0.1 M Tris-buffered saline, pH 7.6, containing 0.3% Triton X-100. Thereafter, slides were incubated overnight at 4 °C with primary antibodies diluted 1:200 in NGS. After several rinses, the sections were incubated at room temperature for 2 h with biotinylated goat anti-mouse or goat anti-rabbit immunoglobulin at the appropriate dilution (Vector Laboratories, Burlingame, CA, USA), followed by 1 h incubation in the avidin-biotin complex (ABC Kit; Vectastain, Vector Laboratories, Burlingame, CA, USA) diluted in tris-buffered saline according to the manufacturer’s instructions and then in 0.05% of 3′-diaminobenzidine (DAB) for 10 min (DAB Sigma Fast, Merck Life Science S.r.l., Milano, Italy). Histological sections were examined under light microscopy with a Leica DMI6000 equipped with Leica DFC340 cooled digital CCD camera (Leica Microsystems, Wetzlar, Germany) and the images were acquired at 10× and 20× magnification [33]. To quantify the density of TNFα-, COX2-, and calnexin-positive signal, each section of AI and MI was divided into three regions (n = 5 animals per treatment; n = 3 pairs of sections/animal, each section selected at 50 μm distance). Digital images were acquired under constant light illumination and magnification, using a digital camera working on gray levels (JCV FC 340FX, Leica Microsystems, Buccinasco MI, Italy). Densitometric analysis of TNFα, COX2, and calnexin peroxidase-based immunostaining was performed by measuring optical density using the image analysis software Image Pro Plus^®^ version 6.0 (MediaCybernetics, Rockville, MD, USA) working on a logarithmic scale of absorbance. In each region, the optical density zero value was assigned to the background (i.e., a tissue portion devoid of stained cells) [33]. Quantitative analysis of mean dead and proliferating cells was performed by counting the caspase 3- or PCNA-positive cells with the nucleus on the focal plane within a box measuring 2 × 104 mm^2^ in the AI or MI (n = 5 animals per treatment; n = 3 pairs of sections/animal, each section selected at 50 μm distance to avoid to count same cells) [34]. All histological analyses were performed by two independent operators blinded to the type of treatment.

### 2.7. Immunofluorescence

For single immunofluorescence, the polyclonal antibody raised in rabbit against ionized calcium-binding adapter molecule 1 (Iba1) (1:200; code 019-19741, WAKO Chemicals, Neuss, Germany) was used. After incubation with the primary antibody, the brain sections were washed several times in PBS and incubated for 2 hours at room temperature with donkey anti rabbit Alexa 488-conjugated secondary antibodies (1:100; Invitrogen, ThermoFisher Scientific, Waltham, Massachusetts, U.S.). Tissue sections were washed in PBS and all slides were coverslipped with Aquatex mounting medium (Merck, Darmstadt, Germany). The immunostained sections were observed with a confocal microscopy Nikon Eclipse Ti2 (Nikon, Florence, Italy) equipped with x-y-z motorized stage, a digital camera DS-Qi2 (Nikon, Florence, Italy), and the acquisition and Image analysis software NIS-Elements C (Nikon, Florence, Italy). Digital images were acquired using the 20–40× objectives. We collected serial Z-stacks of images throughout the area of interest (6–10 planes with an increment varying 0.5–1 μm). Images were deconvolved using the imaging deconvolution software by application of ten iterations. Serial Z plane images were collapsed into a single maximum projection image. Micrographs were saved in TIFF format and adjusted for light and contrast before being assembled on plates using Adobe Photoshop 6.01 (Adobe Systems, San Jose, CA, USA) [33]. The number of cells positive for Iba-1 was determined within a box measuring 2 × 104 μm^2^ that was placed in the lateral, central, and medial areas of hypothalamic nucleus (n = 5 animals per treatment; n = 3 pairs of sections/animal, each section selected at 50 μm distance to avoid to count the same cells). To avoid cell overcounting, only cells with the nucleus on the focal plane were considered [35]. Iba-1-positive cells were identified as resting (with small somata bearing long, thin, and ramified processes) and activated microglia (with hypertrophy together with retraction of processes to a length shorter than the diameter of the somata) or dystrophic microglia. Dystrophic microglia was recognized by debris consisting of several cells displaying fragmented processes and an irregularly shaped cell body as previously demonstrated in humans [35].

### 2.8. Controls

All the antibodies used are reported as reactive for zebrafish. Their specificity was validated with control samples, including: (1) Omission of primary or secondary antibody staining; (2) pre-absorptions of each primary antibody with an excess of the relative peptide (TNFα, and caspase 3) (Figure S1).

### 2.9. Statistical Analysis

The data are expressed as mean ± SEM and analyzed with GraphPad Prism 6 software, version 6.05 (GraphPad, Inc., San Diego, CA, USA). One-way analysis of variance with Bonferroni’s post-hoc test was adopted for the analysis of normally distributed data. For the experiment, including densitometric analysis Kruskal–Wallis Anova nonparametric test followed by Dunn’s post hoc test was adopted for the analysis of non-normally distributed data. A *p*-value < 0.05 was considered as significant.

## 3. Results

### 3.1. 3,5-T2 Increased Body Weight and Serum Triglyceride Levels in D.I.O. Zebrafish

**Figure 1:**
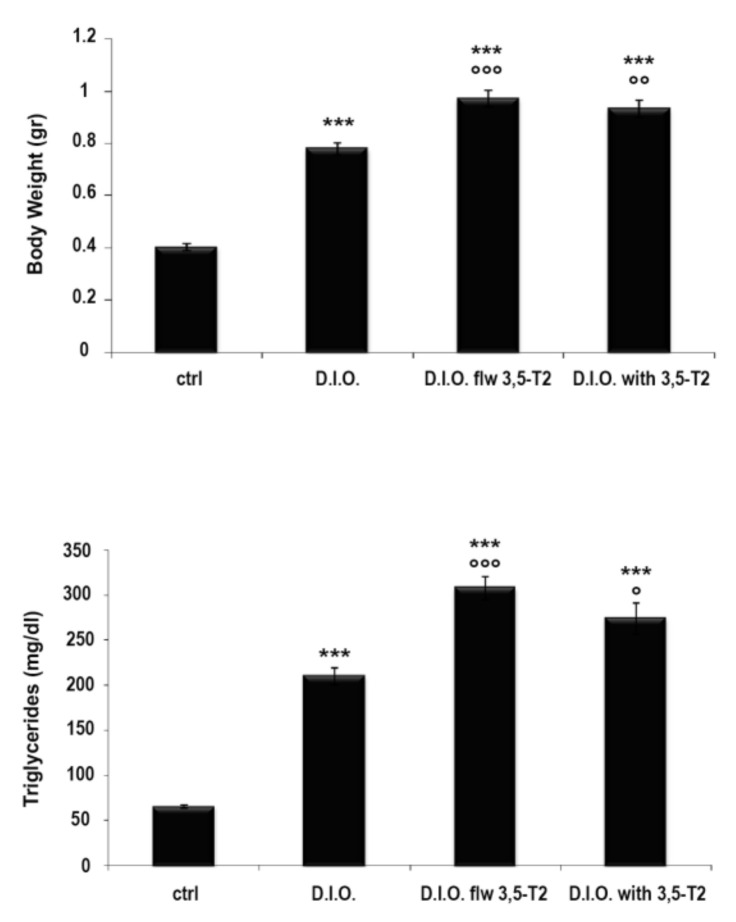
3,5-T2 effect on the body weight and triglyceride levels. ctrl (control zebrafish), D.I.O. (diet-induced obesity zebrafish), D.I.O. flw 3,5–T2 (D.I.O. zebrafish followed by 3,5–T2), D.I.O. with 3,5–T2 (D.I.O. zebrafish treated with 3,5–T2). Data are expressed as mean ± SE. *** *p* < 0.0001 compared to the control group; °°° *p* < 0.0001, °° *p* < 0.0001, and ° *p* < 0.05 compared to D.I.O.

At the end of the experiment significant differences in the body weight were observed among groups. The body weight significantly increased in D.I.O. with respect to the control (ctrl: 0.40 ± 0.013 gr vs. D.I.O.: 0.78 ± 0.021 gr, *p* < 0.0001). The treatment with 3,5-T2 at a concentration of 10 nM caused a significant increase in the body weight both in D.I.O. flw 3,5-T2 (ctrl: 0.40 ± 0.013 gr vs. D.I.O. flw 3,5-T2: 0.97 ± 0.032 gr, *p* < 0.0001) and D.I.O. with 3,5-T2 (ctrl: 0.40 ± 0.013 gr vs. D.I.O. with 3,5-T2: 0.93 ± 0.033 gr, *p* < 0.0001) compared to the control. Moreover, the D.I.O. flw 3,5-T2 and D.I.O. with 3,5-T2 showed an increase in the body weight significantly higher than D.I.O. (D.I.O.: 0.78 ± 0.021 gr vs. D.I.O. flw 3,5-T2: 0.97 ± 0.032 gr, *p* < 0.0001; D.I.O.: 0.78 ± 0.021 gr vs. D.I.O. with 3,5-T2: 0.93 ± 0.033 gr, *p* < 0.001) (Figure 1).

The significant increase in the body weight was accompanied by the increase in serum triglycerides. In particular, D.I.O. zebrafish presented significantly elevated plasma triglyceride levels with respect to the control (ctrl: 65.33 ± 1.84 mg/dL vs. D.I.O.: 209.67 ± 9.22 mg/dL, *p* < 0.0001). The treatment with 3,5-T2 at a concentration of 10 nM caused a significant increase in the triglycerides both in D.I.O. flw 3,5-T2 (ctrl: 65.33 ± 1.84 mg/dL vs. D.I.O. flw 3,5-T2: 307.67 ± 13.04 mg/dL, *p* < 0.0001) and D.I.O. with 3,5-T2 (ctrl: 65.33 ± 1.84 mg/dL vs. D.I.O. with 3,5-T2: 274.11 ± 17.34 mg/dL, *p* < 0.0001), compared to the control. Moreover, the D.I.O. flw 3,5-T2 and D.I.O. with 3,5-T2 showed an increase in triglycerides significantly higher than D.I.O. (D.I.O.: 209.67 ± 9.22 mg/dL vs. D.I.O. flw 3,5-T2: 307.67 ± 13.04 mg/dL, *p* < 0.0001; D.I.O.: 209.67 ± 9.22 mg/dL vs. D.I.O. with 3,5-T2: 274.11 ± 17.34 mg/dL, *p* < 0.05) (Figure 1).

### 3.2. 3,5-T2 Sustained or Increased Intestinal Morphological Alterations Induced by D.I.O.

**Figure 2:**
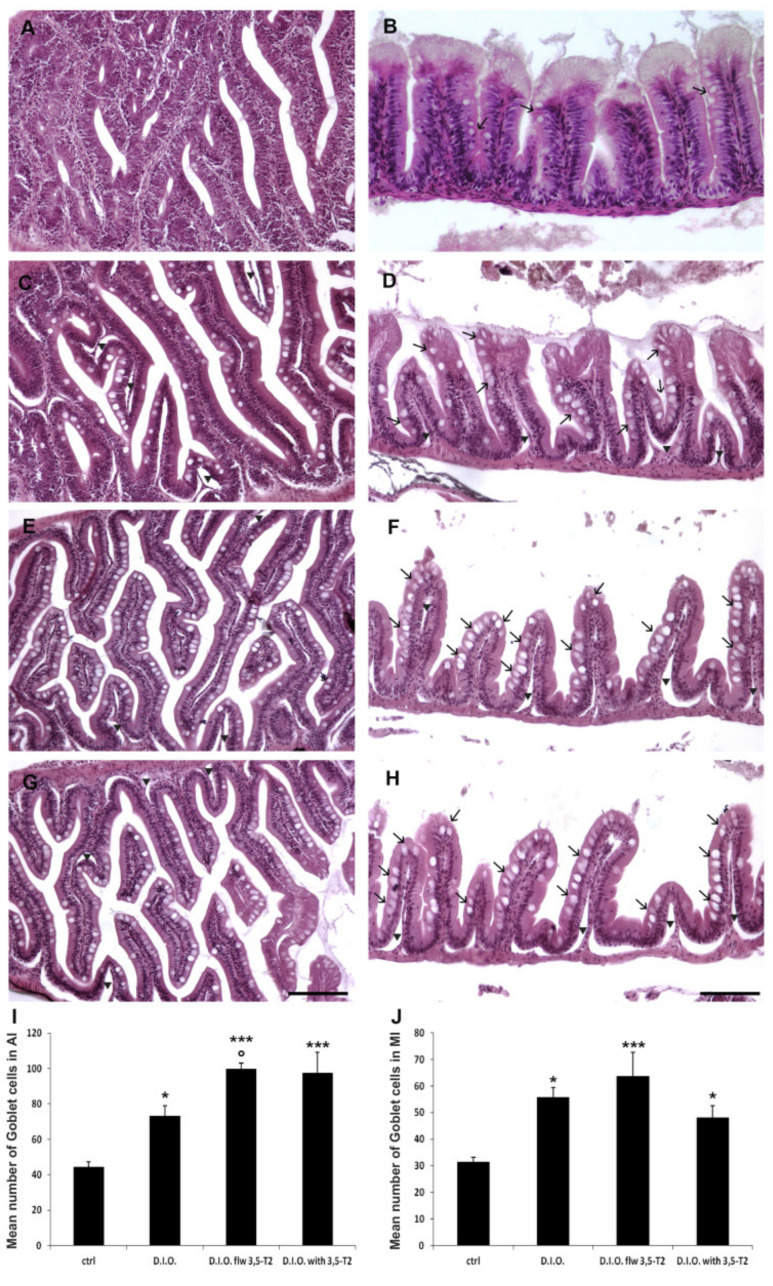
Hematoxylin and eosin (H&E) staining of anterior (AI) and mid (MI) intestine of ctrl (control zebrafish), D.I.O. (diet-induced obesity zebrafish), D.I.O. flw 3,5–T2 (D.I.O. zebrafish followed by 3,5–T2), D.I.O. with 3,5–T2 (D.I.O. zebrafish treated with 3,5–T2). (**A**) AI and (**B**) MI of control zebrafish. (**C**) AI and (**D**) MI of D.I.O. (**E**) AI and (**F**) MI of D.I.O. flw 3,5–T2. (**G**) AI and (**H**) MI of D.I.O. with 3,5–T2. Arrows indicate goblet cells, arrowheads indicate ragged villi. (**I**,**J**) Bar graphs showing the number of goblet cells in the (**I**) AI and (**J**) MI of ctrl, D.I.O., D.I.O. flw 3,5–T2 and D.I.O. with 3,5–T2. Goblet cells were counted based on Alcian Blue staining. Data are expressed as mean ± SE. *** *p* < 0.0001, * *p* < 0.05 compared to the control group. ° *p* < 0.05 compared to D.I.O. Scale bar: 100 μm.

Histological analysis of the intestine is presented in Figure 2. Control zebrafish showed a normal structure of both AI and MI (Figure 2A and B respectively). On the contrary, D.I.O. showed alteration of the intestinal folds (villi) which appeared ragged and irregular both in AI and MI. Debris from ragged villi were detected in the intestinal lumen. In D.I.O., villi alteration was accompanied by a significant increase in the number of goblet cells in AI and MI compared to the control (AI: ctrl: 44 ± 1 vs. D.I.O.: 73 ± 1, *p* < 0.05; MI: ctrl: 31 ± 1 vs. D.I.O.: 55 ± 1, *p* < 0.05). 3,5-T2 treatment led to thinner villi with the consequent enlargement of the gut lumen, together with a significant increase in goblet cell number both in D.I.O. flw 3,5-T2 and D.I.O. with 3,5-T2 with respect to the control zebrafish. More specifically, the quantitative analysis of histological slides stained with Alcian blue, showed a statistically significant increase in the number of goblet cells in the MI of D.I.O. flw 3,5-T2 and D.I.O. with 3,5-T2 (ctrl: 31 ± 1 vs. D.I.O. flw 3,5-T2: 63 ± 3, *p* < 0.0001; ctrl: 31 ± 1 vs. D.I.O. with 3,5-T2: 51 ± 3, *p* < 0.05) and in the AI of D.I.O. flw 3,5-T2 and D.I.O. with 3,5-T2 in comparison to the control (ctrl: 44 ± 0.8 vs. D.I.O. flw 3,5-T2: 100 ± 1, *p* < 0.0001; ctrl: 44 ± 0.8 vs. D.I.O. with 3,5-T2: 98 ± 1, *p* < 0.0001). Moreover, a significant increase in the number of goblet cells was found in the AI of D.I.O. flw 3,5-T2 with respect to the D.I.O. (D.I.O.: 73 ± 1 vs. D.I.O. flw 3,5-T2: 100 ± 1, *p* < 0.05). Although no significant differences were found in the goblet cell number in the AI of D.I.O. with 3,5-T2 and in MI of D.I.O. with 3,5-T2 and D.I.O. flw 3,5-T2 with respect to the D.I.O. zebrafish, they show an increase of morphological alterations with thinner villi and basal membrane disconnection (Figure 2 and Figure S2).

### 3.3. The Cotreatement with 3,5-T2 Sustained Intestinal Inflammation Induced by D.I.O.

**Figure 3:**
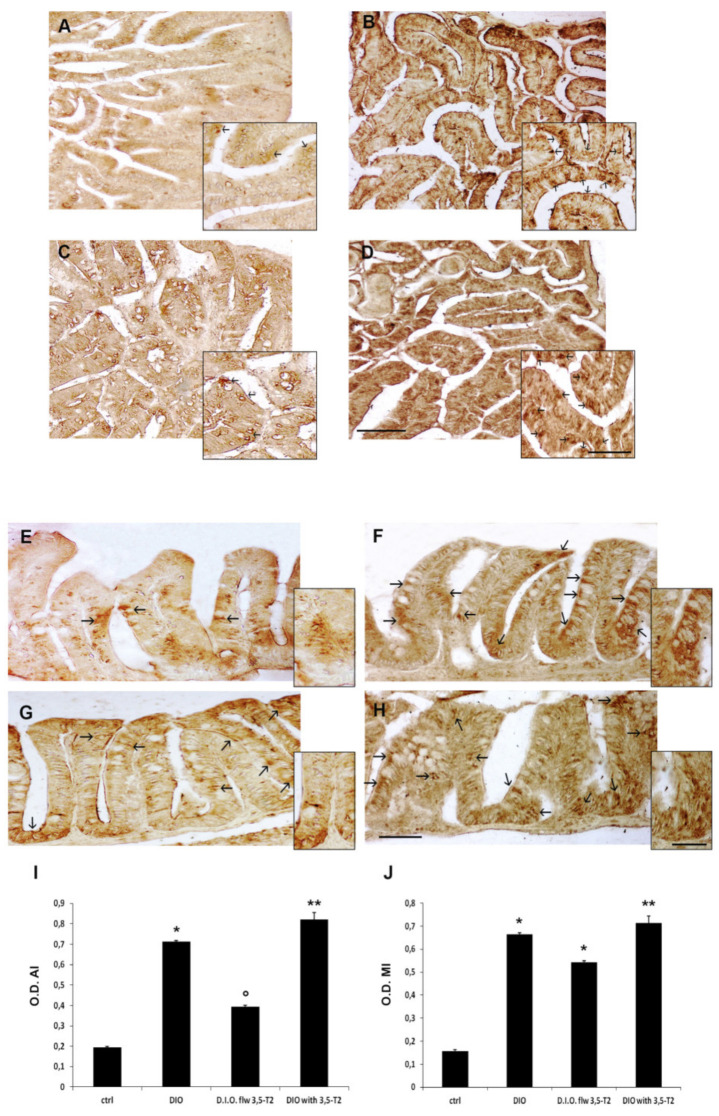
TNFα immunostaining in the anterior (AI) and mid (MI) intestine of ctrl (control zebrafish), D.I.O. (diet-induced obesity zebrafish), D.I.O. flw 3,5–T2 (D.I.O. zebrafish followed by 3,5–T2), D.I.O. with 3,5–T2 (D.I.O. zebrafish treated with 3,5–T2). (**A**) AI of ctrl zebrafish (**B**) AI of D.I.O. (**C**) AI of D.I.O. flw 3,5–T2. (**D**) AI of D.I.O. with 3,5–T2. (**E**) MI of ctrl zebrafish. (**F**) MI of D.I.O. (**G**) MI of D.I.O. flw 3,5–T2. and (**H**) MI of D.I.O. with 3,5–T2. The arrows indicate TNFα immunoexpression in the enteroendocrine and goblet cells. (**I**,**J**) Bar graphs showing TNFα optical density (O.D.) in the (**I**) AI and (**J**) MI of ctrl, D.I.O., D.I.O. flw 3,5–T2 and D.I.O. with 3,5–T2. Data are expressed as mean ± SE. ** *p* < 0.001, * *p* < 0.05 compared to the control group. ° *p* < 0.05 compared to D.I.O. Scale bar: 100 μm in the low magnification and 50 μm in the higher magnification in the boxes of A–D. 50 μm in the low magnification and 25 μm in the higher magnification in the boxes of E–H.

**Figure 4:**
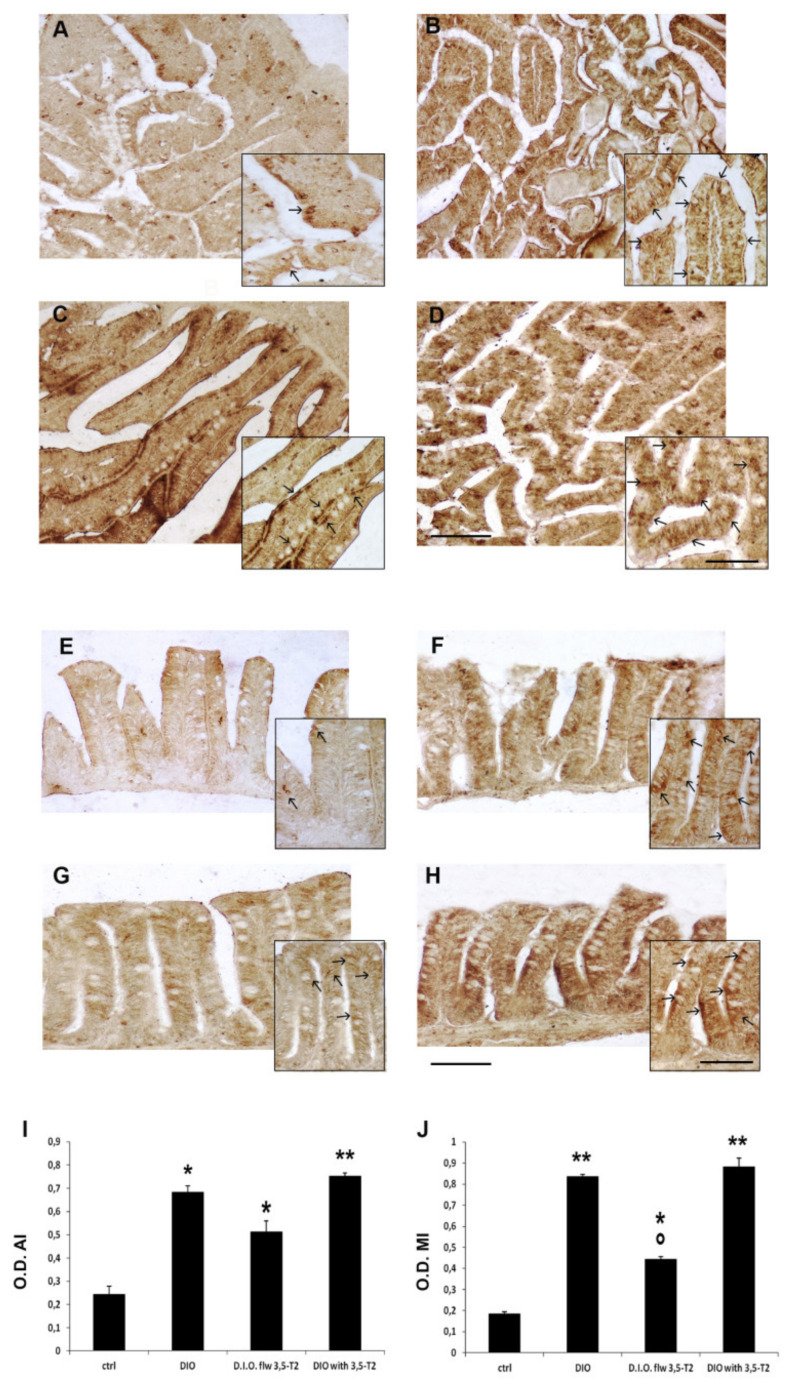
COX2 immunostaining in the anterior (AI) and mid (MI) intestine of ctrl (control zebrafish), D.I.O. (diet-induced obesity zebrafish), D.I.O. flw 3,5–T2 (D.I.O. zebrafish followed by 3,5–T2), D.I.O. with 3,5–T2 (D.I.O. zebrafish treated with 3,5–T2). (**A**) AI of ctrl zebrafish (**B**) AI of D.I.O. (**C**) AI of D.I.O. flw 3,5–T2. (**D**) AI of D.I.O. with 3,5–T2. (**E**) MI of ctrl zebrafish. (**F**) MI of D.I.O. (**G**) MI of D.I.O. flw 3,5–T2. and (**H**) MI of D.I.O. with 3,5–T2. The arrows indicate COX2 immunoexpression in the enteroendocrine and goblet cells. (**I**,**J**) Bar graphs showing COX2 optical density (O.D.) in the (**I**) AI and (**J**) MI of ctrl, D.I.O., D.I.O. flw 3,5–T2 and D.I.O. with 3,5–T2. Data are expressed as mean ± SE. ** *p* < 0.001, * *p* < 0.05 compared to the control group. ° *p* < 0.05 compared to D.I.O. Scale bar: 100 μm in the low magnification and 50 μm in the higher magnification in the boxes.

The morphological alterations of zebrafish intestine induced by D.I.O. and sustained by 3,5-T2 treatment were accompanied by the increased expression of pro-inflammatory markers. In particular, TNFα and COX2 immunoreactivity increased in the enteroendocrine and goblet cells of D.I.O. This increase in TNFα and COX2 immunoexpression in the intestinal cells was generally maintained in D.I.O. with 3,5-T2, showing positivity also in M-like vacuolated cells, while it was partly reduced in D.I.O. flw 3,5-T2 (Figure 3 and Figure 4).

To confirm the increase of TNFα and COX2 immunoexpression in the epithelial cells a densitometric analysis was performed. The densitometric analysis showed a significant increase of TNFα optical density in the epithelium of both AI and MI of D.I.O. with respect to the ctrl (AI: ctrl 0.19 ± 0.01 vs. D.I.O. 0.71 ± 0.009, *p* < 0.05; MI: ctrl 0.15 ± 0.01 vs. D.I.O. 0.66 ± 0.006, *p* < 0.05). A significant increase of TNFα optical density was found also in the MI of D.I.O. flw 3,5-T2 (ctrl 0.15 ± 0.01 vs. D.I.O. flw 3,5-T2 0.54 ± 0.006, *p* < 0.05) and in the AI and MI of D.I.O. with 3,5-T2 (AI: ctrl 0.19 ± 0.01 vs. D.I.O. with 3,5-T2 0.82 ± 0.05, *p* < 0.001; MI: ctrl 0.15 ± 0.01 vs. D.I.O. with 3,5-T2 0.71 ± 0.05, *p* < 0.001) compared to the control. On the contrary, a significant decrease of TNFα optical density was found in the AI of D.I.O. flw 3,5-T2 with respect to D.I.O. zebrafish (D.I.O. 0.71 ± 0.009 vs. D.I.O. flw 3,5-T2 0.39 ± 0.01, *p* < 0.05) (Figure 3).

The inflammatory status was confirmed by COX2 optical density with significant increase in D.I.O. with respect to the control (AI: ctrl 0.24 ± 0.06 vs. D.I.O. 0.68 ± 0.05, *p* < 0.05; MI: ctrl 0.18 ± 0.01 vs. D.I.O. 0.83 ± 0.01, *p* < 0.001). COX2 optical density significantly increased in the AI and MI of D.I.O. flw 3,5-T2 (AI: ctrl 0.24 ± 0.06 vs. D.I.O. flw 3,5-T2 0.51 ± 0.08, *p* < 0.05; MI: ctrl 0.18 ± 0.01 vs. D.I.O. flw 3,5-T2 0.44 ± 0.01, *p* < 0.05) and AI and MI of D.I.O. with 3,5-T2 (AI: ctrl 0.24 ± 0.06 vs. D.I.O. with 3,5-T2 0.75 ± 0.02, *p* < 0.001; MI: ctrl 0.17 ± 0.01 vs. D.I.O. with 3,5-T2 0.88 ± 0.05, *p* < 0.001) with respect to the ctrl. On the contrary, a significant decrease of COX2 optical density was found in the MI of D.I.O. flw 3,5-T2 with respect to the D.I.O. zebrafish (D.I.O. 0.83 ± 0.01 vs. D.I.O. flw 3,5-T2 0.44 ± 0.01, *p* < 0.05) (Figure 4).

### 3.4. The 3,5-T2 Sustained Inflammation Induced by D.I.O. Was Accompanied by ER-Stress in the Anterior and Mid Intestine

**Figure 5:**
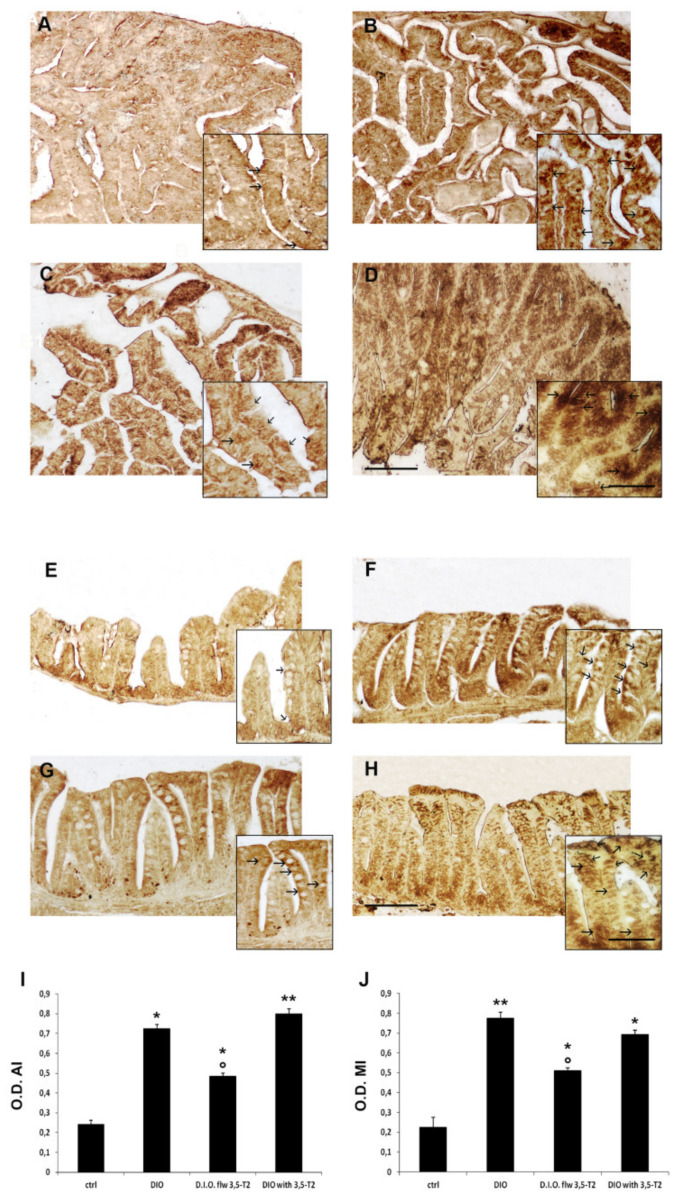
Calnexin immunostaining in the anterior (AI) and mid (MI) intestine of ctrl (control zebrafish), D.I.O. (diet-induced obesity zebrafish), D.I.O. flw 3,5–T2 (D.I.O. zebrafish followed by 3,5–T2), D.I.O. with 3,5–T2 (D.I.O. zebrafish treated with 3,5–T2). (**A**) AI of ctrl zebrafish (**B**) AI of D.I.O. (**C**) AI of D.I.O. flw 3,5–T2. (**D**) AI of D.I.O. with 3,5–T2. (**E**) MI of ctrl zebrafish. (**F**) MI of D.I.O. (**G**) MI of D.I.O. flw 3,5–T2. and (**H**) MI of D.I.O. with 3,5–T2. The arrows indicate calnexin immunoexpression in the enteroendocrine and goblet cells. (**I**,**J**) Bar graphs showing calnexin optical density (O.D.) in the (**I**) AI and (**J**) MI of ctrl, D.I.O., D.I.O. flw 3,5–T2 and D.I.O. with 3,5–T2. Data are expressed as mean ± SE. ** *p* < 0.001, * *p* < 0.05 compared to the control group. ° *p* < 0.05 compared to D.I.O. Scale bar: 100 μm in the low magnification and 50 μm in the higher magnification in the boxes.

The inflammation induced by D.I.O. was accompanied by ER-stress, as indicated by calnexin immunoexpression that increased in the enteroendocrine and goblet cells of D.I.O. This ER-stress was sustained by the cotreatment with 3,5-T2, while the 3,5-T2 post-treatment partly reverted this condition both in the AI and MI. As shown by the densitometric analysis, a significant increase in calnexin optical density was found in the AI and MI of D.I.O. (AI: ctrl 0.24 ± 0.03 vs. D.I.O. 0.72 ± 0.02, *p* < 0.05; MI: ctrl 0.22 ± 0.05 vs. D.I.O. 0.77 ± 0.03, *p* < 0.001), AI and MI of D.I.O. flw 3,5-T2 (AI: ctrl 0.24 ± 0.03 vs. D.I.O. flw 3,5-T2 0.48 ± 0.02, *p* < 0.05; MI: ctrl 0.22 ± 0.05 vs. D.I.O. flw 3,5-T2 0.51 ± 0.02, *p* < 0.05) and in the AI and MI of D.I.O. with 3,5-T2 (AI: ctrl 0.24 ± 0.03 vs. D.I.O. with 3,5-T2 0.80 ± 0.03, *p* < 0.001; MI: ctrl 0.22 ± 0.05 vs. D.I.O. with 3,5-T2 0.69 ± 0.03, *p* < 0.05) with respect to the control. Interestingly, a significant decrease in calnexin optical density was found in the AI and MI of D.I.O. flw 3,5-T2 with respect to D.I.O. (AI: D.I.O. 0.72 ± 0.02 vs. D.I.O. flw 3,5-T2 0.48 ± 0.02, *p* < 0.05; MI: D.I.O. 0.77 ± 0.03 vs. D.I.O. flw 3,5-T2 0.51 ± 0.02, *p* < 0.001) (Figure 5).

### 3.5. The 3,5-T2 Sustained Inflammation Induced by D.I.O. Was Accompanied by Alteration of the Cell Turnover in the Anterior and Mid Intestine

Inflammation and ER-stress induced by D.I.O. and sustained by 3,5-T2 were accompanied by intestinal epithelial apoptosis, as indicated by the increase in caspase-3 immuno-positive cells. In particular, an increase of caspase-3 positive epithelial cells was found in D.I.O., D.I.O. flw 3,5-T2, and D.I.O. with 3,5-T2 respect to the control. An increase of caspase 3 positive cells was observed at the base of the villi, mainly in the AI of D.I.O. and in the MI of D.I.O. with 3,5-T2.

**Figure 6:**
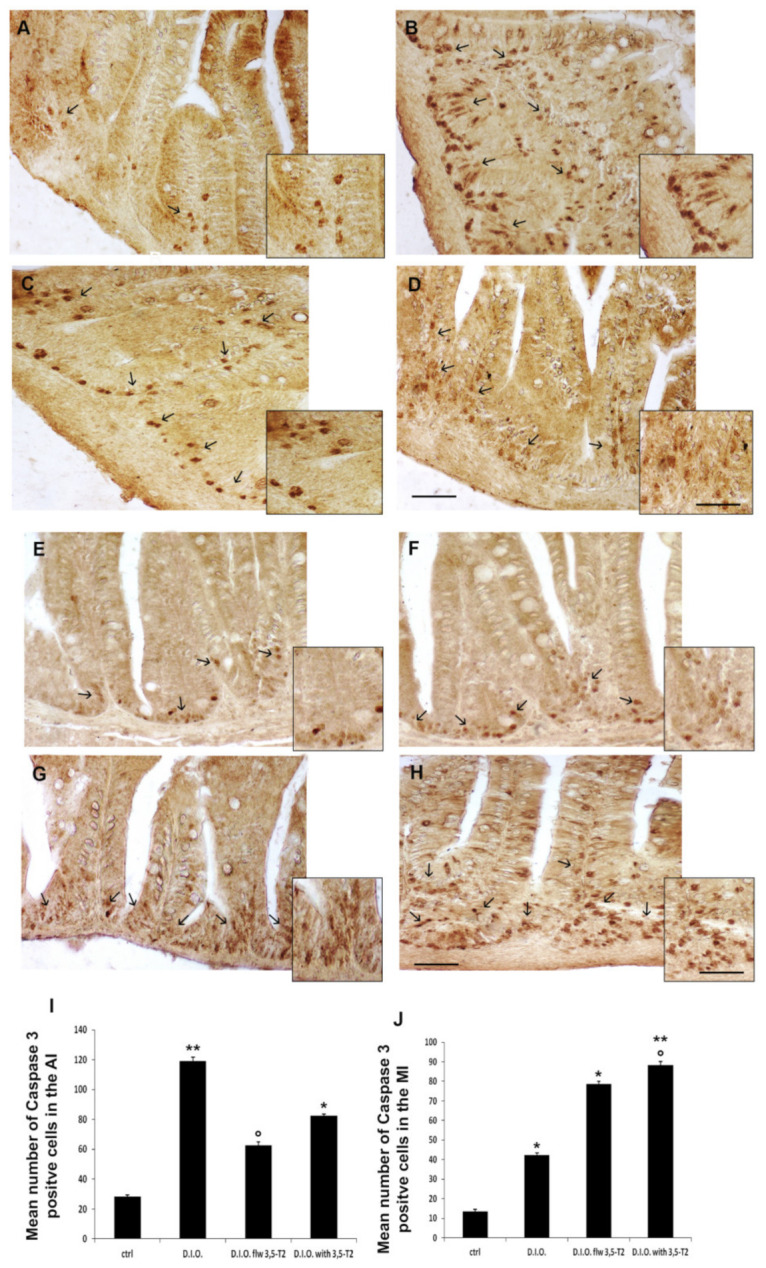
Caspase 3 immunostaining in the anterior (AI) and mid (MI) intestine of ctrl (control zebrafish), D.I.O. (diet-induced obesity zebrafish), D.I.O. flw 3,5–T2 (D.I.O. zebrafish followed by 3,5–T2), D.I.O. with 3,5–T2 (D.I.O. zebrafish treated with 3,5–T2). (**A**) AI of ctrl zebrafish (**B**) AI of D.I.O. (**C**) AI of D.I.O. flw 3,5–T2. (**D**) AI of D.I.O. with 3,5–T2. (**E**) MI of ctrl zebrafish. (**F**) MI of D.I.O. (**G**) MI of D.I.O. flw 3,5–T2. and (**H**) MI of D.I.O. with 3,5–T2. The arrows indicate caspase 3 positive cells in the folds. (**I**,**J**) Bar graphs showing the mean number of caspase 3 positive cells in the (**I**) AI and (**J**) MI of ctrl, D.I.O. flw 3,5–T2, D.I.O. and D.I.O. with 3,5–T2. Data are expressed as mean ± SE. ** *p* < 0.001, * *p* < 0.05 compared to the control group. ° *p* < 0.05 compared to D.I.O. Scale bar: 50 μm in the low magnification and 25 μm in the higher magnification in the boxes.

The cell count showed a significant increase of caspase 3 positive cell number in D.I.O. (AI: ctrl 28 ± 1 vs. D.I.O. 119 ± 3, *p* < 0.001; MI: ctrl 13 ± 1 vs. D.I.O. 42 ± 1, *p* < 0.05), in the MI of D.I.O. flw 3,5-T2 (ctrl 13 ± 1 vs. D.I.O. flw 3,5-T2 78 ± 1.5, *p* < 0.05), and in the AI and MI of D.I.O. with 3,5-T2 zebrafish (AI: ctrl 28 ± 1 vs. D.I.O. with 3,5-T2 82 ± 1, *p* < 0.05; MI: ctrl 13 ± 1 vs. D.I.O. with 3,5-T2 88 ± 2, *p* < 0.001) compared to the control. Interestingly, a significant decrease in caspase 3 positive cell number was found in the AI of D.I.O. flw 3,5-T2 with respect to D.I.O. (D.I.O. 119 ± 3 vs. D.I.O. flw 3,5-T2 63 ± 1, *p* < 0.05), while a significant increase of caspase 3 positive cell number was found in the MI of D.I.O. with 3,5-T2 compared with D.I.O. (D.I.O. 42 ± 1 vs. D.I.O. with 3,5-T2 88 ± 2, *p* < 0.05) (Figure 6).

**Figure 7:**
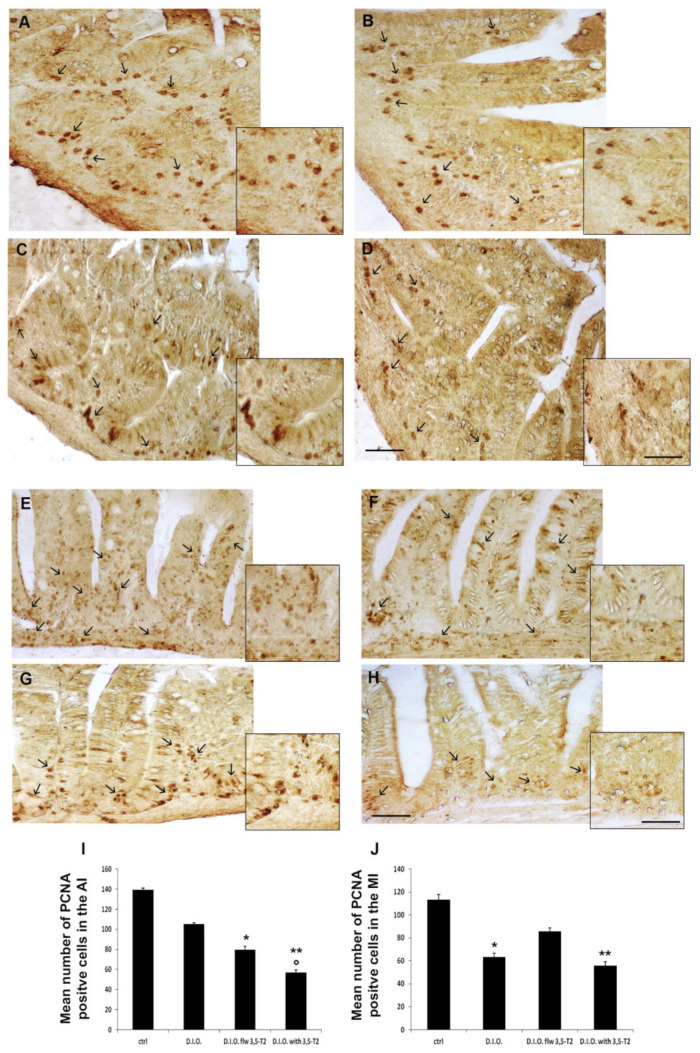
PCNA immunostaining in the anterior (AI) and mid (MI) intestine of ctrl (control zebrafish), D.I.O. (diet-induced obesity zebrafish), D.I.O. flw 3,5–T2 (D.I.O. zebrafish followed by 3,5–T2), D.I.O. with 3,5–T2 (D.I.O. zebrafish treated with 3,5–T2). (**A**) AI of ctrl zebrafish (**B**) AI of D.I.O. (**C**) AI of D.I.O. flw 3,5–T2. (**D**) AI of D.I.O. with 3,5–T2. (**E**) MI of ctrl zebrafish. (**F**) MI of D.I.O. (**G**) MI of D.I.O. flw 3,5–T2. and (**H**) MI of D.I.O. with 3,5–T2. The arrows indicate PCNA positive cells in the folds. (**I**,**J**) Bar graphs showing PCNA optical density in the (**I**) AI and (**J**) MI of ctrl, D.I.O., D.I.O. flw 3,5–T2 and D.I.O. with 3,5–T2. Data are expressed as mean ± SE. ** *p* < 0.001, * *p* < 0.05 compared to the control group. ° *p* < 0.05 compared to D.I.O. Scale bar: 50 μm in the low magnification and 25 μm in the higher magnification in the boxes.

Since the intestine is characterized by a continuous cell turnover, we analyzed also the expression of the proliferative cells. The increase of apoptotic cells was paralleled by the reduction in proliferating cells, as indicated by the decrease in PCNA immuno-positive cells (Figure 7). Specifically, the number of PCNA positive cells was significantly reduced in the MI of D.I.O. with respect to the control (ctrl 113 ± 5 vs. D.I.O. 62 ± 3, *p* < 0.05). The reduction was found also in the AI of D.I.O. flw 3,5-T2 (ctrl 139 ± 2 vs. D.I.O. flw 3,5-T2 79 ± 3, *p* < 0.05), and in the AI and MI of D.I.O. with 3,5-T2 (AI: ctrl 139 ± 2 vs. D.I.O. with 3,5-T2 57 ± 2, *p* < 0.001; MI: ctrl 113 ± 5 vs. D.I.O. with 3,5-T2 55 ± 4, *p* < 0.001) compared to the control. Moreover, a significant decrease in PCNA positive cell number was found in the AI of D.I.O. with 3,5-T2 with respect to the D.I.O. (D.I.O. 105 ± 1 vs. D.I.O. with 3,5-T2 57 ± 2, *p* < 0.05) (Figure 7).

### 3.6. 3,5-T2 Sustained or Increased Brain Inflammation Induced by D.I.O.

**Figure 8:**
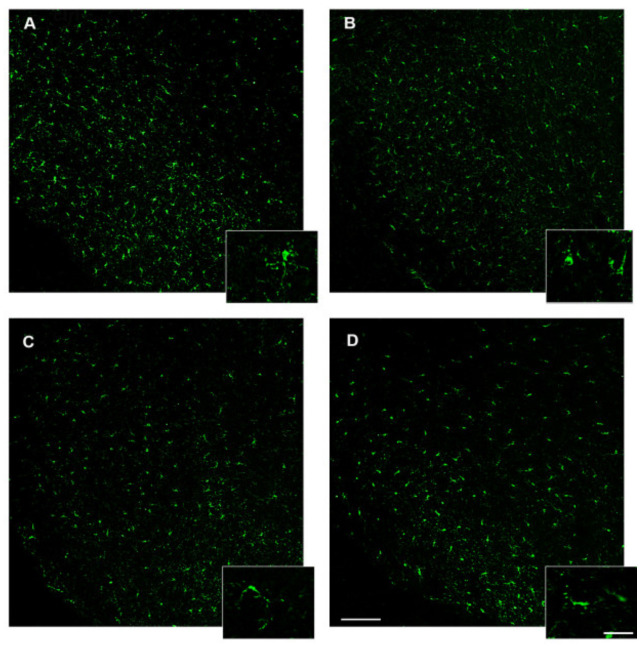
Iba1 immunostaining in the brain of zebrafish. (**A**) Representative image of the hypothalamus of control zebrafish showing resting microglia depicted in the box with high magnification. (**B**) Representative image of D.I.O. (diet-induced obesity zebrafish) hypothalamus showing activated or dystrophic microglia depicted in the box with high magnification. (**C**) Representative image of the hypothalamus of D.I.O. followed by 3,5-T2 showing activated or dystrophic microglia depicted in the box with high magnification. (**D**) Representative image of the hypothalamus of D.I.O. treated with 3,5-T2 showing activated or dystrophic microglia depicted in the box with high magnification. Scale bar: 100 μm in the low magnification and 25 μm in the higher magnification in the boxes.

The peripheral morphological alteration and the activation of the inflammation induced by D.I.O. and sustained by 3,5-T2 were paralleled by the inflammation in the central nervous system. By single immunohistochemistry, we found an increase in Iba1 immunoreactivity coupled with the typical morphology of activated microglia. Microglia, the primary immune cells of the central nervous system, is normally present in the resting state, but it becomes activated under pathological conditions or inflammation state. Iba-1-positive cells were identified as resting (with small somata bearing long, thin, and ramified processes) in the control group, and activated (with hypertrophy together with retraction of processes that appeared shorter than the diameter of the somata) or dystrophic microglia (with fragmented processes and an irregularly shaped cell body) in D.I.O., D.I.O. flw 3,5-T2 and D.I.O. with 3,5-T2 (Figure 8).

**Figure 9:**
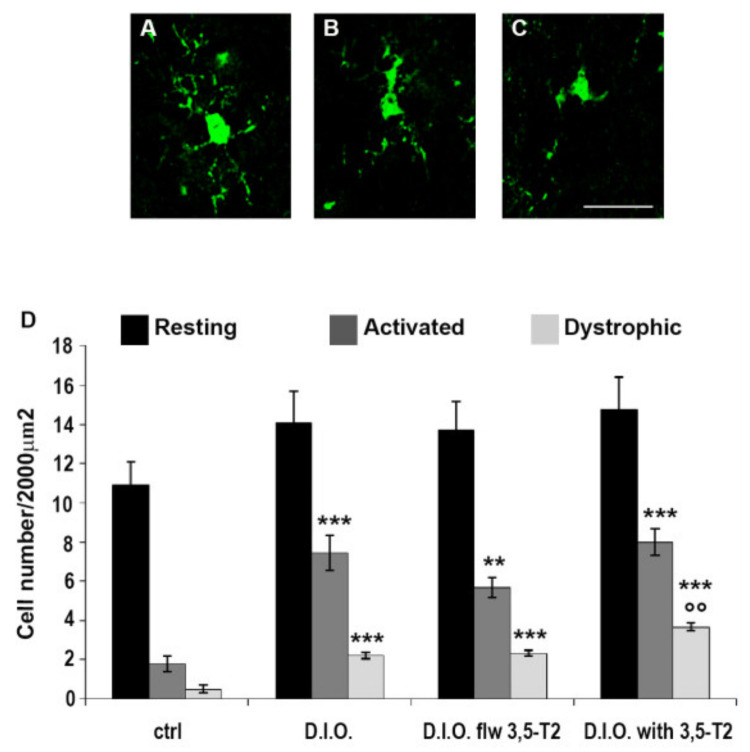
Morphological analysis of microglia in the brain of zebrafish. (**A**–**C**) Representative images of different microglial morphology. (**A**) resting microglia, (**B**) activated microglia, and (**C**) dystrophic microglia. Scale bar: 25 μm. (**D**) Quantitative analysis of the resting, activated, and dystrophic-like cells in the hypothalamus of ctrl (control zebrafish), D.I.O. (diet-induced obesity zebrafish), D.I.O. flw 3,5–T2 (D.I.O. zebrafish followed by 3,5–T2), D.I.O. with 3,5–T2 (D.I.O. zebrafish treated with 3,5–T2). Data are expressed as mean ± SE. *** *p* < 0.0001, ** *p* < 0.001 compared to the control group. °° *p* < 0.05 compared to D.I.O.

In particular, the quantitative analysis of resting, activated, or dystrophic microglia in the hypothalamus of D.I.O. zebrafish showed a significant increase of activated microglia with respect to the control (ctrl: 1.78 ± 0.4 vs. D.I.O.: 7.44 ± 0.9, *p* < 0.0001), with a significant increase of dystrophic microglia (ctrl: 0.5 ± 0.2 vs. D.I.O.: 2.2 ± 0.17, *p* < 0.0001). Moreover, the D.I.O. flw 3,5-T2 and D.I.O. with 3,5-T2 also have showed a significant increase of activated microglia (ctrl: 1.78 ± 0.4 vs. D.I.O. flw 3,5-T2: 5.67 ± 0.5, *p* < 0.0001; ctrl: 1.78 ± 0.4 vs. D.I.O. with 3,5-T2: 8 ± 0.67, *p* < 0.0001) and dystrophic microglia (ctrl: 0.5 ± 0.2 vs. D.I.O. flw 3,5-T2: 2.33 ± 0.13, *p* < 0.0001; ctrl: 0.5 ± 0.2 vs. D.I.O. with 3,5-T2: 3.67 ± 0.19, *p* < 0.0001) compared to the control. More interestingly, the D.I.O. with 3,5-T2 showed a significant increase in the number of dystrophic microglia with respect to the D.I.O. (D.I.O.: 2.2 ± 0.17 vs. D.I.O. with 3,5-T2: 3.67 ± 0.19, *p* < 0.0001) (Figure 9)

## 4. Discussion

3,5-T2 is well-known for its role in promoting metabolic rate and oxygen consumption, leading to beneficial metabolic effects on HFD rodents [20,36]. Goglia and colleagues have shown the positive role of 3,5-T2 on mitochondrial functions, fat mass, and lipid metabolism, supporting the use of 3,5-T2 as a drug for the treatment of obesity [14,37,38]. Although the ability of 3,5-T2 to increase metabolism has been reported [39], the safe and effective dose as well as the possible adverse effects of this molecule have not been sufficiently explored. Moreover, a lack of studies exists on the possible effects of 3,5-T2 on the intestine and the brain.

Given the highly conserved gut function and immunity-related genes between mammals and zebrafish, the latter has become an interesting animal model to investigate the essential processes underlying intestinal inflammation and injury [40]. Recent studies have revealed a well-conserved organization of the intestinal system and brain between zebrafish and mammals [29,41,42]. Moreover, strong evidence supports that the overconsumption of nutrients leads to systemic inflammation and alters metabolic homeostasis acting on central and peripheral systems [3,43]. Previous studies have shown the increase in intestinal inflammation in D.I.O. zebrafish [1,3,6], but no data on the role of 3,5-T2 are available.

Here we show the 3,5-T2 functional role in peripheral and central inflammation in D.I.O. zebrafish model. We detected that the dose of 3,5-T2 can affect not only the health status, but also the survival of zebrafish. In particular, our results indicate that a high dose of 3,5-T2 (100 nM) caused death, while a lower dose (10 nM) did not affect survival, although increased the morphological alterations in the intestine caused by D.I.O, and sustained the peripheral and central inflammation. The high mortality induced by the 100 nM dose could be explained by the reported thyrotoxic effect induced by chronic administration of high doses of 3,5-T2 in mice [21].

The study presented here demonstrates that both the post- and contemporary- treatment with 3,5-T2 (10 nM) of D.I.O. zebrafish, strongly affected weight and triglyceride levels. This first result is in contrast with previous studies which report a positive or null effect of 3,5-T2 on weight and fat mass in high fat fed rodents [39,44].

Studies in humans and animal models, such as rodents and zebrafish, report cases of intestinal inflammation induced by a HFD [45,46,47]. Such alterations are induced and modulated by pro-inflammatory cytokines, that are well-known to play a key role in metabolic diseases [10]. 3,5-T2 is associated with many modern lifestyle-induced conditions, and zebrafish has emerged as an important model for human endocrine diseases including obesity, diabetes, and metabolic syndrome [48]. In this study, 3,5-T2 treatment led to a worsening of the intestinal morphological alterations caused by a high-fat diet. In particular, in the D.I.O. zebrafish, the mucosal architecture was damaged, as revealed by (i) the cell fusion at the apical border of the villi, (ii) the thinning of the villi themselves and iii) the significant increase of goblet cell number. The post- or contemporary-treatment with 3,5-T2 of D.I.O. zebrafish sustained these morphological alteration and in particular increased the number of goblet cells in the anterior intestine, which is the first gut segment providing the nutrient absorption.

However, the post- and contemporary- treatment with 3,5-T2 (10 nM) of D.I.O. zebrafish showed different effect on the activation of pro-inflammatory markers, with the contemporary treatment sustaining the effect of D.I.O. and the post-treatment partially reverting the effect of D.I.O. Mainly, our results are in disagreement with the beneficial role of 3,5-T2 reported in many studies [14,19,36], since the comparative analysis of D.I.O. vs. 3,5-T2-treated D.I.O. zebrafish indicates a role for 3,5-T2 in the upregulation of pro-inflammatory markers. In this study, 3,5-T2 co-treatment of D.I.O. zebrafish generally sustained the increase of pro-inflammatory markers, such as TNFα and COX2, two major mediators of inflammation. On the contrary, the post-treatment with 3,5-T2 of D.I.O. zebrafish induced a decrease in the expression of these inflammatory markers. The mitochondrium is one of the cellular targets of 3,5-T2, where it stimulates the respiration via a direct action on the cytochrome C oxidase (COX), with a consequent reduction of the respiratory efficiency [13,14,15,16]. 3,5-T2 leads to a rapid increase in mitochondrial oxygen consumption, which is then reflected at the whole animal level [17]. The increase of COX expression is one of the major markers of inflammation and, interestingly, in our study the contemporary treatment of D.I.O. zebrafish with 3,5-T2 sustained the enhancement of COX2 expression induced by D.I.O. This effect could be due to the alteration of mitochondrial number and oxidative function induced by chronic HFD and which can change with type of fat contained in the diet inducing obesity [49,50].

This inflammatory pathway activated by D.I.O. and maintained by 3,5-T2 induced cellular stress, as confirmed by the significant increased expression of calnexin, a marker of alteration of the endoplasmic reticulum activity (ER-stress) in intestinal enteroendocrine and goblet cells. On the contrary, the post-treatment with 3,5-T2 reduced the activation of this inflammatory pathway, as confirmed by the decrease of calnexin expression.

Often inflammation and ER-stress are accompanied by apoptotic events. In agreement with the inflammation status, 3,5-T2 co-treatment of D.I.O. zebrafish caused the increase of caspase 3-immunoexpression in the mid intestine. Since it is well-known that in the zebrafish intestine a constant cell turnover is at the basis of the correct morphology and activity of the villi [40], we analyzed also the number of proliferating cells. As expected, the enhancement of apoptotic cells was paralleled by the 3,5-T2 sustained decrease in proliferating cells, which can explain the reduction of villi size with the consequent enlargement of the gut lumen.

Therefore, the contemporary treatment with 3,5-T2 of D.I.O. zebrafish did not ameliorate the inflammation status induced by diet, on the contrary, it seemed to sustain the high expression of markers that play a substantial role in the modulation of inflammation. However, the post-treatment with 3,5-T2 of D.I.O. zebrafish partially reverted the inflammation status induced by the high-fat diet, maybe because of the association of 3,5-T2 treatment with a change of diet. Thus, the use of 3,5-T2 as pharmacological agent and diet supplement should be considered with caution, taking into consideration that it is a metabolic derivate of thyroid hormones, in turn, able to exert side effects on target organs, such as intestine, liver, kidney, and heart. Moreover, while most of the studies reporting beneficial effects of 3,5-T2 have been performed in rodent models of obesity with chronic hypothyroidism [51,52,53,54], data on the effects of 3,5-T2 on metabolism in HFD euthyroid animal models or obese models brought back to a normal diet are scarce [55]. In a recently published study, the treatment with 3,5-T2 performed in diet-induced obese euthyroid mice, resulted in the reduction of body fat mass, but with an undesired negative feedback inhibition of the hypothalamus–pituitary–thyroid (HPT) axis accompanied by increased heart weight [20].

In this study, the 3,5-T2-sustained inflammation seemed to occur in both AI and MI, when 3,5-T2 treatment was given at the same time of the obesity-inducing diet, while when given after D.I.O. and in association with a normal diet it partly reverted the inflammation status. The intestine is one of the primary organs responsible for the absorption of nutrients and can be modified by the diet. Through the well-known bi-directional gut–brain axis, the intestine can induce changes in the central nervous system and consequently affect the brain functions [56,57]. We show here that the contemporary treatment with 3,5-T2 negatively modulated the expression of pro-inflammatory markers supporting the alteration of the intestine morphology and the activation of inflammation in the gut induced by D.I.O. Such pro-inflammatory effect was accompanied by an increase of inflammation in the central nervous system. Indeed, we detected a strong microglia activation in the brain of D.I.O. zebrafish treated with 3,5-T2, with an increase of dystrophic microglia in numerous brain regions, such as the hypothalamus, which is the main feeding center of the central nervous system. Therefore, we can hypothesize that the co-treatment with 3,5-T2 during obesity inducing diet, led to a reinforcement of the pro-inflammatory pathways at peripheral level and consequent intestine morphological alteration, able to induce an increase of triglyceride levels and to sustain inflammation at central nervous system level through the gut-brain axis. These side effects probably affect nutrient absorption and the central regulation of food intake, leading to the increase in the body weight and triglycerides.

To the best of our knowledge, this is the first immunohistochemistry analysis of intestine and brain tissues from D.I.O. zebrafish treated with 3,5-T2. Densitometric and quantitative approaches showed strong effects of 3,5-T2 on body weight and triglycerides accompanied by a sustained peripheral and central inflammation in D.I.O. zebrafish. These findings are surprising since they are in contrast with previous studies in HFD rodents reporting beneficial effects of 3,5-T2 on the metabolic rate [14,58]. However, our results are in agreement with the conclusions of recent studies on 3,5-T2 action in rat hepatic nuclei [28,59], where 3,5-T2 showed toxic effects in the liver [60]. We should take into account that the effects of 3,5-T2 can be influenced by the model, the dietary conditions, the diet fat composition, and by 3,5-T2 concentration, uptake, metabolism, and elimination. Overall, administration of 3,5-T2 might exert or sustain peripheral and central adverse effects induced by diet and additional studies are required to further delineate the mechanisms whereby 3,5-T2 acts and the specific contexts in which it can be efficacious or toxic. Therefore, the potential use of 3,5-T2 to fight obesity, as it is suggested in several publications or commentaries, should be viewed with caution, posing special attention to the role played by the diet associated with 3,5-T2 treatment.

## 5. Conclusions

Since it has been reported that the thyromimetic effects of 3,5-T2 treatment may affect many organs, resulting in long-term undesirable effects, a reliable model to study the side effects of 3,5-T2 on important organs targeted by thyroid hormones (e.g., pituitary, brain, gut, heart, bone, muscle) is clearly useful to evaluate any potential risk. Zebrafish has been recognized as an excellent animal model for studying human metabolic and inflammatory diseases. We suggest that the adult zebrafish is a reproducible and adequate model to test the effects of 3,5-T2 on the gut–brain axis, with particular attention to the systemic and central inflammation induced by the diet. This work underlies how the zebrafish model can help to better understand the fundamental beneficial and side effects of 3,5-T2, which is of great importance to define the possible use of this metabolite of thyroid hormones as a drug in different diseases including obesity.

## Supplementary Materials

The following are available online at https://www.mdpi.com/2076-2615/10/7/1131/s1, Figure S1: Antibody specificity control. Figure S2: Alcian blue staining of the zebrafish intestine.
