# Supplementary material for: Immunohistochemical Analysis of Intestinal and Central Nervous System Morphology in an Obese Animal Model (Danio rerio) Treated with 3,5-T2: A Possible Farm Management Practice?
Source: Animals (Basel). 2020 Jul 3;10(7):1131. doi: 10.3390/ani10071131 (PMC7401507; doi:10.3390/ani10071131)
Supplement: Supplementary file 1 [file animals-10-01131-s001.pdf]

Supplementary Materials

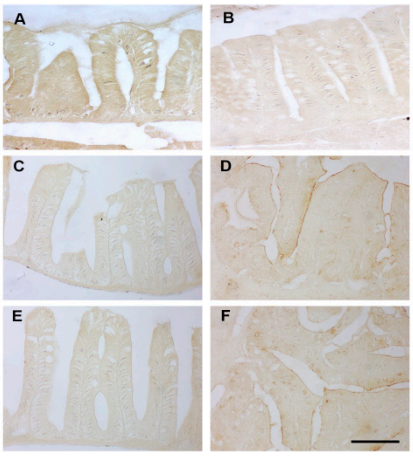

Figure 1. Antibody specificity control. (A) Control of TNFα with pre-absorptions of the primary antibody with an excess of the relative peptide. (B) Control of caspase 3 with pre-absorptions of the primary antibody with an excess of the relative peptide. (C, D) Representative images of the specificity of the antibodies COX2 and Calnexin validated by omitting the primary antibodies. (E, F) Representative images of the specificity of the antibody PCNA validated by omitting the primary antibody. Scale bar: 100 µm.

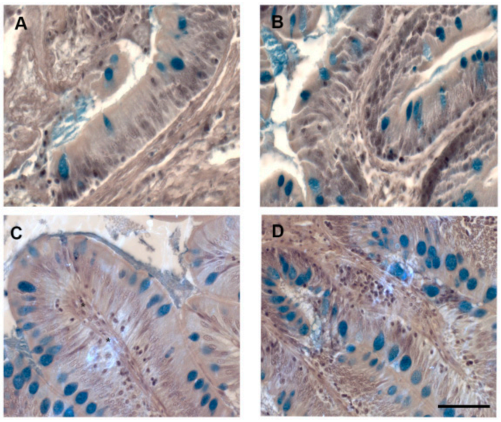

Figure 2. Alcian blue staining of the zebrafish intestine. (A) Anterior intestine (AI) of control zebrafish. (B) AI of Diet induced obesity (D.I.O.) zebrafish. (C) AI of D.I.O. zebrafish followed by 3,5–T2. (D) AI of D.I.O. zebrafish treated with 3,5–T2. Scale bar: 25 µm.
